# Preservation of Hearing and Facial Nerve Function with the Microsurgical Excision of Large Vestibular Schwannomas: Experience with the Retrosigmoid Approach

**DOI:** 10.7759/cureus.3684

**Published:** 2018-12-04

**Authors:** Muhammad Shaheryar Ahmed Rajput, Ahmad Nawaz Ahmad, Asif Ali Arain, Mohammad Adeel, Saeed Akram, Muhammad Sohail Awan, Muhammad Ehsan Bari

**Affiliations:** 1 Otolaryngology, Liaquat University of Medical and Health Sciences, Karachi, PAK; 2 Otolaryngology, Liaquat National Hospital and Medical College, Karachi, PAK; 3 Otolaryngology, The Indus Hospital, Karachi, PAK; 4 Otolaryngology, Shaukat Khanum Memorial Cancer Hospital and Research Center, Lahore, PAK; 5 Internal Medicine, King Faisal Specialist Hospital & Research Center, Riyadh, SAU; 6 Surgery, Aga Khan University Hospital, Karachi, PAK; 7 Neurosurgery, Aga Khan University Hospital, Karachi, PAK

**Keywords:** vestibular schwannoma, acoustic neuroma, retrosigmoid craniotomy, retrosigmoid approach, hearing preservation, cerebellopontine angle, schwannoma

## Abstract

Introduction

Vestibular schwannomas (VS) are the most common benign neoplasms of a cerebellopontine angle (CPA), which arise from the Schwann cells of the vestibulocochlear nerve. Eighty percent of CPA tumors are VS followed by meningioma as the second common mass lesion in this critical potential space. Treatment options range from watchful waiting with serial imaging studies to radiosurgery or a microsurgical excision or a combination of surgery and radiation therapy. The primary objective of the study was to assess hearing and facial nerve status before and after the surgery via the retrosigmoid approach.

Methods

The database of Aga Khan University Hospital was searched for diagnoses of vestibular schwannomas between 2000 and 2007. A total of 35 patients were identified; among them, 27 were selected for the study who met the inclusion criteria. The variables of the study were age, gender, presenting symptoms, size of the tumor, surgical approach, hearing levels, and facial nerve function. Hearing loss was categorized according to the Gardener-Robertson hearing classification and the House-Brackmann Scale was used for facial nerve assessment.

Results

Out of the 27 patients, 18 were male and nine were female. The mean age was 43 years. The most common presenting complaint was hearing loss and tinnitus, seen in 21 patients. Headache was present in six patients, ataxia in five, and vertigo in three. Facial nerve weakness was noticed in six patients. Two patients had Grade-III paralysis, three had Grade-IV paralysis, and one had Grade-V paralysis. The audiogram confirmed the presence of sensorineural hearing loss (SNHL) in all patients. Twelve patients out of 27 had Class II hearing with the threshold between 31 and 50 decibels and a Speech Discrimination Score (SDS) of 50% to 69%. Ten patients had non-serviceable hearing and the remaining five had poor hearing. The audiogram was repeated after surgery for those 12 patients who had Class II hearing and showed that seven out of 12 patients maintained a hearing threshold within the range of Class II at the one-year follow-up (hearing preservation 58%). The facial nerve preservation rate was 56% considering House-Brackmann Grade III or less as acceptable facial nerve function.

Conclusion

The optimal treatment for small vestibular schwannomas is a matter of controversy; however, the choice of treatment for large vestibular schwannomas in patients without significant comorbidity is generally microsurgical excision. The surgical excision of a large VS with the retrosigmoid approach is found to be safe consistently. The hearing and facial nerve preservation in our study were found comparable with the literature.

## Introduction

Vestibular schwannomas (VS) are benign neoplasms that arise from the myelin sheath of the eighth cranial nerve. They account for 8% of all intracranial tumors and 80% of cerebellopontine angle tumors [1]. Treatment options include microsurgery, fractionated stereotactic radiotherapy, stereotactic radiosurgery, and a combination of surgery and radiotherapy or observation [2]. The goals of VS management have shifted from surgical excision to functional preservation, particularly when the removal of the tumor is not considered safe in terms of cranial nerve preservation [3-4]. Studies have shown that the function of the facial nerve is compromised in attempts of gross total removal of a large VS [5-6]. Depending on many factors, including patient age, tumor size, growth, and symptomatology, patients can choose surgery, radiation, or conservative management [7].

More than 90% of the cases are unilateral and present at a median age of 50 years. Patients usually present with unilateral hearing loss and tinnitus. Pure tone audiometry reveals asymmetric sensorineural hearing loss. Loss of speech discrimination score is out of proportion to the hearing impairment and this prompts further investigation with magnetic resonance imaging (MRI) of the internal auditory canal. Symptomatology is a function of cranial neuropathies that result due to direct pressure as the tumor grows. The average growth rate of vestibular schwannomas ranges from 2 mm to 4 mm per year in 50% of the cases, whereas less than 10% can regress [8]. The extramural tumors are more likely to grow than those located within the internal auditory canal [9]. Tumor expansion in the cerebellopontine angle causes a compression of the brainstem and leads to life-threatening neurological complications.

Generally, small to medium-sized tumors that are less than 3 cm can be managed differently from the large vestibular schwannomas. Surgery is the favored option over stereotactic radiation therapy for large vestibular schwannomas. Some of the authors reported the successful treatment of a large VS with stereotactic radiation therapy [10]. However, others believe that radiation therapy may risk ischemia of the seventh cranial nerve and brainstem compression with the consequent complications [11-12]. The optimal treatment, particularly for a small to medium-sized tumor, remains controversial and the choice of modality of treatment remains at the discretion of the treating physician and available resources. Therefore, the approach to the patient is variable from center to center. The symptomatic younger patients, particularly those with persistent dizziness, with anatomically favorable tumors and a good hearing level, are offered gross total resection and also those with larger tumors and a mass effect on the brain stem [12].

The preservation of hearing and facial nerve function has always been an important consideration in the management of VS. The current study was conducted at the Aga Khan University Hospital, which is a tertiary care referral center in the city of Karachi, Pakistan. The primary objective of the study was to assess hearing and facial nerve status before and after the surgery via the retrosigmoid approach.

## Materials and methods

The database of Aga Khan University Hospital (AKUH) was searched for the diagnosis of vestibular schwannoma between 2000 and 2007. Total 35 patients were identified among them 27 were selected for the study and the remaining eight patients were excluded because they either had previous surgery outside AKUH or radiation therapy or the data required for the study was not adequately available.

Review of the medical records including physician’s notes, imaging study and audiograms were carried out. The variables of the study were age, gender, presenting symptoms, size of the tumor, surgical approach, hearing levels, and facial nerve function. The hearing loss was categorized according to the Gardener-Robertson hearing classification, as shown in Table 1 [13].

**Table 1:** Gardener-Robertson classification of hearing

Class	Description	Audiogram	Speech Discrimination Score
I	Good/excellent	0 – 30	70% – 100%
II	Serviceable	31 – 50	50% – 69%
III	Non-serviceable	51 – 90	5% – 49%
IV	Poor	91 max	1 – 4%
V	None	Not testable	0

Preoperative and postoperative facial nerve status were assessed with the House-Brackmann grading system as shown in Table 2 [14].

**Table 2:** House-Brackmann scale

SCALE	Description
Grade I	Normal
Grade II	Noticeable on close inspection
Grade III	Obvious but not disfiguring
Grade IV	Disfiguring
Grade V	Barely perceptible movement
Grade VI	Complete paralysis

## Results

Out of 27 patients, 18 were male and nine were female. The mean age was 43 years. The most common presenting complaint was hearing loss and tinnitus, seen in 21 patients. Headache was present in six patients, ataxia in five, and vertigo in three. Facial nerve weakness was noticed in six patients. Two patients had Grade-III paralysis, three had Grade-IV paralysis and one had Grade-V paralysis. Twenty-one patients had normal facial nerve function at presentation. Clinical presentation is shown in Table 3.

**Table 3:** Clinical presentation of the patients

Clinical Feature	Number of Patients
Hearing Loss	21
Tinnitus	21
Headache	6
Ataxia	5
Vertigo	3
Facial Palsy	6

The audiogram confirmed the presence of sensorineural hearing loss (SNHL) in all patients. SNHL was also seen on audiogram in those patients who did not report a hearing loss at the time of presentation. According to the Gardner-Robertson hearing classification, 12 patients out of 27 patients had serviceable hearing with a hearing threshold between 31 and 50 decibels and Speech Discrimination Score (SDC) 50% to 69%. Ten patients had a non-serviceable hearing and remaining five had poor hearing. All patients were investigated with contrast-enhanced MRI, which revealed large vestibular schwannoma in the cerebellopontine angle. The size of the tumor was reported between 3 cm to 4 cm in six patients and more than 4 cm in 21 patients.

All patients were treated surgically with the retrosigmoid craniotomy approach by neurosurgeons. The audiogram was repeated after surgery for those 12 patients who had preoperative serviceable hearing (Class II). The postoperative audiogram showed seven out of 12 patients maintained a hearing threshold within the range of Class II at the one-year follow-up (hearing preservation 58%). Study variables and results are shown in Table 4.

**Table 4:** Variables and results

Variables	Results
Gender	Male = 18, Female = 9
Mean age	43 years
Preoperative serviceable hearing	12 patients
Postoperative serviceable hearing	7 patients
Tumor size 3 to 4 cm	6 patients
Tumor size >4 cm	21 patients

The facial nerve function assessment at the one-year follow-up was noted as Grade I in five patients, Grade II in three patients, Grade III in five patients, Grade IV in 10 patients, and Grade V in four patients. Preoperative and postoperative facial nerve status is shown in Table 5.

**Table 5:** Preoperative and postoperative facial nerve function according to the House-Brackmann grading scale

	Grade I	Grade II	Grade III	Grade IV	Grade V
Preoperative	21	0	2	3	1
Postoperative	5	3	5	10	4

The facial nerve preservation rate was 56% considering House-Brackmann Grade III or less as acceptable facial nerve function.

## Discussion

The management of vestibular schwannomas is a matter of debate, particularly for small tumors that are less than 2 cm in size. The larger and symptomatic tumors are generally considered for microsurgical excision except in cases with significant comorbidities complicating open surgery [15]. The utmost goal of surgery for vestibular schwannomas in patients with serviceable hearing is to achieve complete resection with the preservation of hearing [16-18].

The retrosigmoid approach for vestibular schwannomas provides panoramic visualization of the cerebellopontine angle from the tentorium cerebelli to the foramen magnum. A retraction of the temporal lobe of the brain is not required in the retrosigmoid approach. The main disadvantage is the limited exposure of the internal auditory canal, hence, the lateral dissection of the tumor is difficult [19]. The likelihood of hearing preservation becomes less if the tumor infiltrates the cochlear nerve, preoperative hearing is poor, or the tumor size is large.

In recent years, some skull base surgeons started the use of endoscopes for the excision of the tumors in the cerebellopontine angle. These endoscopic-assisted procedures are now gaining popularity for the posterolateral skull base surgery [20].

The size of the tumor does not necessarily correlate with the presence of serviceable hearing at the time of initial presentation [21]. This is also seen in 12 of our patients who had serviceable hearing despite large grade 4 tumor size according to the Koos classification [22].

Injury to the facial nerve is one of the major complications with the surgical management of vestibular schwannomas [23]. Facial nerve function is considered satisfactory as long as the House-Brackmann Grade is 3 or less. The review of 89 articles on facial nerve function reported 78% of preservation with the retrosigmoid approach in tumor size of <20 mm and 67% in larger tumors [24]. The facial nerve preservation rate in our study, where all patients had large tumors, was 56% considering House-Brackmann Grade III or less as acceptable facial nerve function.

All patients were operated with the retrosigmoid approach in the standard position, as described in the literature [25-26]. Significant variations have been reported regarding hearing preservation after microsurgical resection of large vestibular schwannomas, ranging from 11% to 70% [27-28]. In a study of 54 patients, preservation of hearing was reported in 53.7% of patients with a size of more than 3 cm vestibular schwannomas [29]. In our study, we found 12 patients with serviceable hearing preoperatively and after surgery, seven out of those 12 patients had a serviceable hearing at the one-year follow-up. The hearing preservation rate in our study was 58%, which is comparable to the percentages reported in the literature for large vestibular schwannomas. The highest rate of a hearing preservation was reported in the middle cranial fossa approach - as high as 70% in small tumors, which decreases to 50% as the tumor size increases [30].

## Conclusions

The optimal treatment for the small vestibular schwannoma is a matter of controversy; however, the choice for the treatment of a large vestibular schwannoma in patients without significant comorbidity is generally microsurgical excision. Surgical excision of a large VS with the retrosigmoid approach is consistently found safe. The hearing and facial nerve preservation in our study were found comparable with that in the literature.
